# Sport culture and communication among middle school athletes, parents, and staff: A qualitative study

**DOI:** 10.1371/journal.pone.0282252

**Published:** 2023-03-15

**Authors:** Zachary Yukio Kerr, Paula Gildner, Stephanie K. Parker, Vasiliki Kostogiannes, Christine E. Callahan, Aliza K. Nedimyer, Melissa K. Kossman, Avinash Chandran, Johna K. Register-Mihalik

**Affiliations:** 1 Department of Exercise and Sport Science, University of North Carolina at Chapel Hill, Chapel Hill, NC, United States of America; 2 Injury Prevention Research Center, University of North Carolina at Chapel Hill, Chapel Hill, NC, United States of America; 3 Department of Physical Therapy, College of Pharmacy & Health Sciences, Campbell University, Buies Creek, NC, United States of America; 4 Physician Assistant Program, University of Maryland-Baltimore, Baltimore, MD, United States of America; 5 Human Movement Science Curriculum, University of North Carolina at Chapel Hill, Chapel Hill, NC, United States of America; 6 School of Health Professions, University of Southern Mississippi, Hattiesburg, MS, United States of America; 7 Datalys Center for Sports Injury Research and Prevention, Indianapolis, IN, United States of America; Mugla Sitki Kocman University: Mugla Sitki Kocman Universitesi, TURKEY

## Abstract

Middle school (MS) is an intermediary level of education between elementary and secondary school that typically includes students aged 10–15 years. There is limited research within the MS sport setting, particularly related to sport-related injury prevention. This qualitative study aimed to better understand the sport culture within MS sports and the communication strategies used among invested groups (i.e., athletes, parents, staff inclusive of coaches and school nurses). Semi-structured interviews were conducted with 19 athletes, 20 parents, and 18 staff (e.g., coaches, school nurses) from seven MS in two school districts during the 2018/19 and 2019/20 school years. Topics focused on understanding school- and sport-related factors related to education, safety, and communication. Analysis used a consensual qualitative research tradition, in which the research team discussed individually developed themes and categories from transcribed interviews, with the goal of coming to a consensus and creating a codebook. Throughout the coding process, the research team would reconvene to discuss coding decisions until consensus was reached. This study focuses on the themes of sport culture and communication. Dominant categories identified within sport culture related to participants noting why they were interested in MS sports, and their struggles with their perceived roles and engagement (e.g., helping parents stay engaged, finding coaches, oversight of school nurses). Competitiveness and safety could have conflicting roles and priority. Dominant categories identified within communication centered around limited communication between coaches and parents. Technological assistance (e.g., phone apps, websites) was available, but often varied by school and sport. Concussions were seldom discussed unless during the preseason meeting or when one occurred. Findings highlight that the MS sport settings may struggle with incorporating primary prevention into their cultures and ensuring reliable communication among individuals. Novel and tailored approaches to injury prevention are needed to help ensure buy-in and proper implementation.

## Introduction

Youth sports participation has been associated with physical, psychological, social, and academic benefits [1]. However, participation in such activities can also lead to injury and related adverse long-term outcomes [2–5]. Much discussion about injury in youth sports is centered around concussion, with an estimated 1.1–1.9 million sport- and recreation-related concussions sustained among youth ≤18 years in the United States (US) [6]. As such, organizations such as the Aspen Institute have called for an emphasis on injury prevention to help encourage and maintain sports participation among youth [7].

As injury prevention is considered across youth sports, there has been a growing interest in United States (US) middle school (MS) sport settings. MS is an intermediary level of education between elementary and secondary school that typically includes 3 to 4 grade levels (ranging from 5th to 9th grades) and students aged 10–15 years. This level may also be referred to as intermediate school or junior high. Previous injury incidence estimates, particularly for concussion, in MS sports are comparable to those of high school and youth league settings [8, 9], but have been limited in sample size, focused on specific schools or school districts. Nonetheless, the findings have substantiated hypotheses related to the role of unique structures and factors that may be associated with injury risk and management, including smaller team sizes, athletes being multi-sport athletes, and limited access to medical care [8, 9]. There is limited research within the MS sport setting, particularly related to sport-related injury prevention and management.

Previous research among athletes older than the MS age range (e.g., high school and college) note the role of teammate influence in injury reporting, particularly in regard to concussion [10–12]. However, given the age and structure of MS environments, several groups of individuals may play a role in the sport environment and medical care-seeking. Current data suggest youth athletes are highly influenced by parents and coaches/staff [13]. Specifically, parents influence youth athletes’ motivation, perceived achievement in sports, and perceptions of fair play [13–15]. Furthermore, they also make the healthcare decisions of their children [16, 17]. Coaches are fundamental in creating a team culture in which injury care-seeking are perceived as important [18–22]. These individuals are especially salient in the context of concussion [23–27]. However, additional staff such as school nurses may also be instrumental in the provision of concussion-related care. To best understand injury prevention and resultant intervention strategies aimed at reducing injury burden in MS settings, the identification and understanding of its unique structures, influential factors, and overall sport culture and communication among these groups of individuals is warranted.

Given the limited research within the MS sport setting, the primary aim of the current study is to better understand the sport culture within MS sports and the communication strategies used among invested groups (i.e., athletes, parents, staff inclusive of coaches and school nurses). Given concerns about concussion in youth settings [28–31], the study includes a focus on concussion-related risk and safety among athletes.

## Materials and methods

This study is part of a larger project focused on developing an intervention to help individuals within MS sport settings (e.g., athletes, parents, staff) better communicate concussion-related prevention and management information to their peer networks [16]. The study was approved by the Institutional Review Board at the University of North Carolina at Chapel Hill. All participants provided informed written consent. The study also abided by the Standards for Reporting Qualitative Research [32].

In the current study, semi-structured interviews were conducted with athletes, parents, and staff (e.g., coaches, school nurses) from seven MS in two school districts from one county in a southeastern state during the 2018/19 and 2019/20 school years (**Table 1**). Sports sponsored within these two districts (but not necessarily within each school) included: Baseball, Basketball, Cheerleading, Cross-Country, Field Hockey, Football, Lacrosse, Soccer, Softball, Tennis, Track, Ultimate Frisbee, Volleyball, and Wrestling.

**Table 1:** Sociodemographics of students at middle schools.

	Number students enrolled	Percentages		
		Female students	Students of color	Students on free/ reduced lunch
District 1				
School 1	694	50	42	25
School 2	654	51	47	24
School 3	668	49	52	26
School 4	817	46	48	30
District 2				
School 5	483	46	51	19
School 6	640	46	59	21
School 7	649	48	31	15

NOTE: Data represent sociodemographics of all students within districts (and not solely those participating in current study).

### Study population and sample recruitment

The population of interest was MS athletes, parents, and staff. The sample was selected from in-person recruitment efforts in which the research team provided study information at: fall, winter, and spring sport parent meetings; sport-specific parent meetings; and sport-specific games and practices. When recruiting, the research team discussed the lack of research in the MS setting and the study’s intent to better understand sport injury prevention and management.

To be eligible to participate, athletes had to be enrolled at one of the seven MSs during the 2018/19 or 2019/20 school year and on a roster for a school-sanctioned sport; parents had to have a child fitting the previously listed athlete criteria; and staff had to be employed at one of the seven MSs during the 2018/19 or 2019/20 school year and either serve as a school nurse or a coach for a school-sanctioned sport. Staff could be comprised of employees of the school that had more than one role (e.g., teacher that are also coaches) or individuals who were contracted to coach a sport exclusively (e.g., community member volunteering to coach). Recruitment continued until saturation was reached. It is also important to note that interviews were conducted prior to the start of the COVID-19 pandemic.

**Fig 1:**
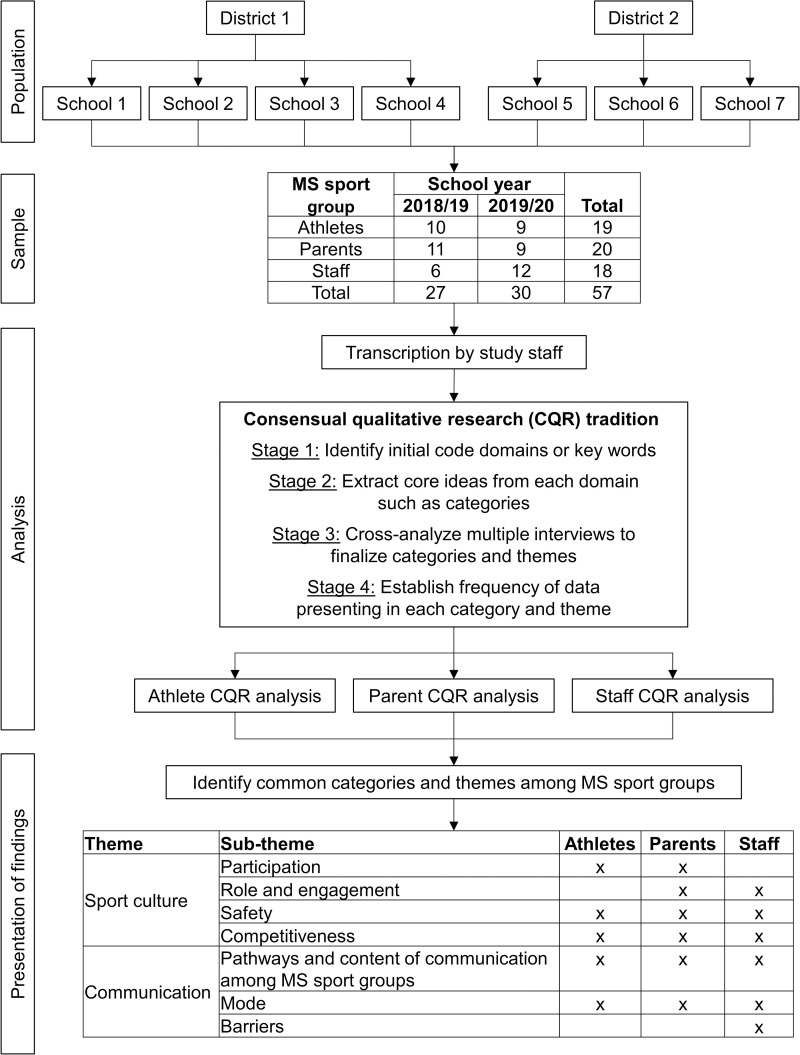
Study recruitment, analysis, and themes/categories identified.

Overall, 57 participants (athlete n = 19, parent n = 20, staff n = 18) had interviews conducted either in-person or via phone (Fig 1). The age range of the 19 athletes was 12–14 years. Of the 14 athletes providing gender and race/ethnicity information, 57.1% (n = 8) identified as male; 64.3% (n = 9) identified as White/non-Hispanic and 35.7% (n = 5) identified as Black/African American. The median age of the 20 parents was 44 years (range of 33 to 60), with 75% (n = 15) identifying as female; 80.0% (n = 16) identified as White/non-Hispanic and 20.0% (n = 4) identified as Black/African American. The median age of the 18 staff was 28 years (range of 19 to 52). Of the 14 staff providing gender and race/ethnicity information, 78.6% (n = 11) identified as male; 92.9% (n = 13) identified as White/non-Hispanic and 7.1% (n = 1) identified as Black/African American.

### Data collection

Three members of the research team served as the interviewers. The first was a White/non-Hispanic woman with a public health background and a multiracial MS-aged child participating in youth sports. The second and third were both White/non-Hispanic women that had experience as athletic trainers and with concussion rehabilitation. (The remaining research team was a multi-racial/ethnic group of researchers from public health and/or athletic training that specialized in injury and concussion prevention-related research). The interviewers had no prior relationship with study participants before study commencement. Only the interviewer and participant were present during interviews. No repeat interviews were conducted.

At the beginning of the interview, participants provided written consent to participating in the study and being audio-recorded. Prior to obtaining written consent from minors, written consent was also obtained from their parents. The interviewer followed a semi-structured interview protocol (Table 2). Topics focused on understanding school- and sport-related factors related to education, safety, and communication. For each MS sport group, questions were modified to ensure understandability (e.g., simplified for athletes, specific to MS regulations for staff). In addition, the interviewer was encouraged to ask follow-up questions where deemed fit to gain further information.

**Table 2:** Content of semi-structured phone interview with middle school athletes, parents, staff.

Content	Included in specific sport group’s interview protocol?		
	Athlete	Parent	Staff
General (e.g., how did you/your child get involved in sports?)	X	X	X
School-related factors (e.g., What is a typical day like for you with middle school sports?)		X	X
Middle school sport regulation (e.g., There are policies and rules about sport safety and concussion. What do you think are the benefits and consequences of having these in place?)			X
Sport safety and concussions (e.g., What comes to mind when you hear the word “concussion”?)		X	
Education for sport safety and concussions (e.g., What motivates you to talk about concussions with others?)	X	X	X
Communication within sport (e.g., As a parent, how involved are you in your child’s team?)	X	X	X
Education and information (e.g., What did you learn about concussions in your sport this past season?)	X	X	X
Closing (e.g., Does anyone have any other topics to talk about or final comments/suggestions before we end?)	X	X	X

The development and refinement of the interview protocol was conducted with the aid of disciplinary experts. Additionally, we conducted pilot testing of the interview protocol with a convenience sample of two athletes, three parents, and one coach. The interview protocol was revised based on feedback prior to deployment. The resulting audio recordings were reviewed and transcribed by a member of the research team. To protect confidentiality, identifying information related to the participants and their schools were edited out of all materials.

### Analysis

Analysis used a consensual qualitative research (CQR) tradition, which originates from a grounded theory and phenomenological approach (Fig 1). These approaches help to explore novel issues that have not be heavily researched previously. The CQR tradition consisted of four progressive stages. Stage 1 identified initial code domains or key words. Stage 2 extracted core ideas from each domain to create categories. Stage 3 cross-analyzed multiple interviews to finalize categories. Each codebook for each group was created independently. Stage 4 established the frequency of data presenting in each category and theme.

This process used a multi-person research team and relied on the process of consensus. The team was comprised of four coders and one auditor. First, all interviews were transcribed. Next, for each MS sport group (i.e., athletes, parents, staff), five interviews were selected by the research team for their diversity in discussion points. The research team then individually coded for Stages 1 and 2. The research team reconvened to discuss their individually developed themes and categories, with the goal of coming to a consensus and creating a codebook. The research team then coded the remaining interviews individually according to the consensus codebook. Throughout this process, the research team would reconvene as necessary to discuss coding decisions until consensus was reached. Although each codebook for each MS sport group was created independently, themes and relevant content are presented together given the similarities among MS sport groups. Finally, to aid trustworthiness of findings, the following results text was reviewed multiple times by the interviewers and CQR teams to ensure depictions presented represented what they perceived to be accurate.

## Results

Although multiple themes were identified among the constituency groups, for this paper, we are focusing on the themes of sport culture and communication (Fig 1). There was overlap amongst the identified categories and sub-categories among all three groups. Given such similarities among MS sport groups (i.e., athletes, parents, and staff), categories and relevant content are presented together. Quotes embedded in the manuscript text noting the specific group represented by the participant and their study ID number, e.g., “(Athlete #12022),” “(Parent #11056).” Although themes were identified in the context of school staff, quotes are noted as originating from either coaches or school nurses, as applicable.

### Sports culture

Sports culture considered the overarching culture related to playing sports within MS settings. Identified categories were related to participation, competitiveness, roles, and safety. Table 3 provides exemplar quotes related to the categories and sub-categories.

**Table 3:** Categories identified in interviews with middle school athletes, parents, and staff related to the category of “sport culture”.

Category	Example quotes
Participation *Athletes (n = 18/19)* *Parents (n = 18/20)* *Not a category for staff*	*Reasons why athletes want to participate* “My dad was always really big on things like baseball and football and all that. And though my parents were concerned about safety issues, I just kind of got into it really quickly and then continued it.” (Athlete #12022) “A lot of my friends were doing it and I wanted to do sports and so I decided to do that.” (Athlete #12034) *Reasons why parents want their children to participate* “…comradery that I think a team provides has really helped him navigate some of the tumultuous transitions of adolescence (Parent #11056)” “the environments are really around the development of the child, not necessarily of the development of the athlete” (Parent #21023) “his mother and I really wanted to make sure that sort of physical activity was part of how he knew the world to be, so we started him playing soccer when he was probably like 3” (Parent #11056)
Role/Engagement *Parents (n = 19/19)* *Staff (n = 18/18)* *Not a category for athletes*	*Staying engaged as parents* “School soccer, they practice every day after school except for Friday. But actually their games are during the week…We don’t even see him until after 5:00! … And then he’s also doing club soccer which is practices are actually three times a week in the evening. So, when we had both, it was crazy … he comes home, has something quick to eat and then goes to the next one.” (Parent #11019) “If you have a parent who steps up to be sort of an administrative person who can stay on top of that, the coach can reach out to you, for example, that works better. So, we’ve had a variety of experiences with that.” (Parent #31015) *Finding coaches* “They were from the community, either from [nearby university] or someone who worked in the area or whatever. Sometimes it’s a parent that comes and becomes a coach. So they search for coaches not just within the school system but throughout because I think sometimes they have a hard time finding coaches too.” (Parent #31020) “Most times you’re going to want to have a teacher who works in the school to be a coach at the same time. It’s mostly for discipline reasons I’d say more than anything else. Just if there’s any problems with a kid with academics or acting out in class then the teacher is going to reach out to you and you can take them away from the sporting event or whatever, talk to them or make them have some sort of consequence for their actions. At the school right now though I think we do a lot of coaches from the community and [nearby university] coaches. I know our Lacrosse team had girls who were on the club team at [the nearby university]. I know that our Track coach was a [nearby university] student. Sometimes it’s hard to get those positions filled. I know our athletic director tries to make sure it’s within the school but he has to use what he can get. I mean we almost lost the Lacrosse team because we couldn’t get a coach until the very last minute. We got a good Lacrosse coach though.” (Staff/Coach# 23003) *Ensuring safety and education* “teacher first, coach second” (Staff/Coach# 13003) “I worry so much about educating the children. But it’s if there’s an accident, you’re putting attention all on that child over the next 24 hours, 36 hours. And we timeframe to make sure that they didn’t get injured or have a concussion. Um, during practice, you’re just looking for safety stuff. You’re not going to say “hey, be careful you can get a concussion from this”. You can’t live like that. But just making sure that safety operations are in place, any sort of dangers or…you’re taking care of that beforehand so that hopefully they don’t occur.” (Staff/Coach# 73003) “I think a lot of that would fall on the athletic director to make sure that all of the coaches in that school are aware of all of the requirements. All of the training, all of their certification, taking online classes, or whatever. So, I think it comes from…the culture needs to be established up top and fall down.” (Staff/Coach# 53005) *The limits as a school nurse* I’m a nurse and I’m not allowed to diagnose. So I’d never say “I think they have a concussion” because that’s medical practice and I can’t do that. So, I would say what their symptoms are, if the coach came to me, and “I’d strongly suggest and recommend that you go get medical attention.” That’s what I would say. (Staff/Nurse #14001) Our job as nurses is not to monitor to make sure people are doing the report. Our job is to make sure that these kids are OK and they’re going back OK into healthy and injury-free as possible… (Staff/Nurse #34001) I’m treating symptoms…And even when I have documentation, as a nurse I’m still treating symptoms. I’m not an M.D. that can do much beyond that. We’re following what the M.D.s are saying to do. (Staff/Nurse #44001)
Competitiveness *Athletes (n = 19/19)* *Parents (n = 19/20)* *Not a category for staff*	*Competitiveness of youth sports* “over time…sports have gotten more competitive for kids, a lot more competitive, even like club sports” (Parent# 31020). “I think it’s as competitive as the NFL! I mean they’re out there, they want to be the team, they want to win. I’m always amazed at whatever age it was they were interested in winning than anything else” (Parent# 21006) “The school thing is very competitive in the sense that it’s sort of bragging rights … people want to win and at the end of the whole thing, one team wins the whole thing…But then club is like a totally different cup of tea. It’s like there’s different levels. There’s silver, gold, bronze and then you try out, you get placed at whatever level. And then they go out and play all different types of games, sometimes tournaments in other states and things like that. So, in some ways it’s more competitive” (Parent# 11019) “it wasn’t really fun to like be there socially because everybody was just focused on…practice, practice, practice” (Athlete# 12051) *Focusing on fun over competitiveness* “I really like how competitive [sports] are, which can get dangerous at points I suppose. But I don’t know, it’s a way to get your emotions out and a way to work out I guess. (Athlete# 12002)” “Oh, we were horrible. We won two games each season. It was just us having fun and to keep moving.” (Athlete# 32015) “Cross-country is like everybody is a winner. I mean sometimes the kids will have like “team pride.”… the girls and boys run separately but they stay and cheer each other on. It’s the opposite of competitive even against the other schools, like their rivals. They all know them.” (Parent# 21009) “Ultimate [Frisbee] was like “This is fun.” I don’t know. The sport dynamic, you could feel it. It was different … Like the kids were still smiling and cheering each other on. I don’t know how to explain it.” (Parent# 11022) *Competitiveness putting athletes at risk for injury* “[Competitiveness] could lead to a situation even in a safe sport like cross-country where you can get a concussion” (Parent# 31017) “I think a lot of it is the ‘walk it off’ attitude. It’s a lot of the time what you need to do; just get up and keep going and you’re fine. You might hurt for a minute.” (Parent# 31004) *Role of coaches and schools to monitor sports* “I think any coach has a big role in that. It’s kind of like a leader in a work situation because they set the tone. I mean we’ve been very fortunate with both my kids to have a lot of coaches who are very positive. It’s about bringing a positive spirit. It’s not about winning. But there’s also, I’ve seen a lot of coaches that aren’t. And that really trickles down pretty fast. It sets the tone for the kids. It shows what they are focusing on. It shows even what skills and drills they’re doing. So, I think who the coaches are and how they lead is critical. And I think that’s really important. I think that if you have the same coach and you play the same sport, in middle school if you’re playing for two years or three years, if you have the same coach then they know you already so there’s continuity which can be really nice in building relationships.” (Parent# 31020) “[My school] does a pretty good job of letting them determine how competitive they’re going to be. And that’s good. We had a couple of really—- I don’t even know if “competitive” would be as good a term as just “interested.” Especially one of the really gifted athletes on the team, the one parent was just really into it. But yet at the same time it was more about the son than it was about winning or anything like that.” (Parent# 11002)	
Safety *Athletes (n = 19/19)* *Parents (n = 20/20)* *Staff (n = 18/18)*	*Perspective on injuries* “The parents are a bit more concerned about like the tangible injuries, like torn ACL’s, broken ribs, broken arm, broken leg. The injuries that you can’t see, no one talks about…Because you can’t see it” (Parent# 41003) “You’re going to get hurt eventually sometime in the season on way or another.” (Athlete# 12002) *Changing culture* “just a lot more about prevention and speaking…speaking to [athletes] about, like what we talked about—the rebounding techniques. And things to avoid concussions. In the past, I guess we were aware of them, if they happened. But we weren’t super active or proactive about preventing them. And so, I think the prevention aspect has definitely increased a lot.” (Staff/Coach# 43003) “So, you know, we like to continue to enhance that and share that with the other schools we work with. And make sure the district is, you know, focused on those efforts as well. And hopefully that district, our district, is you know, sharing what they know with other districts and it just spreads. Or we’re getting information from other districts that are doing things a little bit better, so we are on the same page and always promoting the welfare of the student-athlete.” (Staff/Coach# 53003) *The role of all individuals within MS sports* “Like when, at the very beginning when we like made the team we like sat down and we talked about a lot of things like what we’re going to do in practice and one of those things was like make sure we don’t get hurt and like and we talked about concussions there and stuff.” (Athlete# 22023) “Well, our coaches, they watch pretty carefully. They’re like ‘I want to see if anyone is covering something up because it’s not as important to me that we win as your safety.’” (Athlete# 32015) “Yeah. So, I mean I think that a sport safety culture you know does come from a coach, but also from the school and kind of the community … I guess I would say that as a parent it’s also you know, my responsibility to—kind of be checking in with my kid and you know during coaching orientation—and like parent orientation to be kind of listening for safety type messages.” (Parent #21023) “I think parents definitely have a role to play—In sort of having the expectation that, you know, safety is the priority over winning and really over anything else. As parents we should demand that.” (Parent# 11056) “I think the refs needs to take the forefront on that. And the refs need to be more receptive to the coaches. Because the coaches are seeing…you know, the refs are looking for wherever the ball is for that call. Whereas, coaches are watching everything. … And parents should be able to let either coach or the ref know without assuming that they are taking on too much of a…they’re being too crazy of a spectator.” (Staff/Coach# 73003) *The role of policy and rules* “Just the basic rules like when the whistle blows the play stops, with no more hits or anything. There’s a certain set, like there’s like a certain limit where if you do something too bad like hit, like hit too dirty or have like an attitude, they’ll throw a flag.” (Athlete# 42003) “Like be respectful of other people’s space I guess. When you’re playing soccer there’s rules like you can’t high kick where your cleat goes too high in the air because there have been times where like people get a cleat in the mouth or in the head and that can cause concussions or losing teeth.” (Athlete# 12022) “I think that if players know they’ll get a foul call on them if they do do something dangerous, then they’ll be less likely to do it.” (Athlete# 22006) “some schools [changed] how they practice to reduce the occurrence of concussions during practice” (Parent# 11045). “I’ve hardly ever seen it. It’s just like a different, like it’s not in the game. When the kids know that they can’t head it they just don’t. I can’t even think of a time where a kid headed a ball and they blew the whistle. I’m almost certain that in school, because I go to all the school games and all that, I don’t think there’s any heading at all.” (Parent# 11019) “So, if we suspect a student has a concussion we have to report it right away to the athletic director and to the nurse and possibly the principal just to keep them in the loop. Once we do that, we go through the concussion protocol and they can’t come back.” (Staff/Coach# 13003) *Issues with implementation* “I think that a lot of the coaches now are if there’s concern about it they’re taking them out. They’re monitoring. They’re asking the questions initially, having them sit it out for a little bit, continuing to monitor them. If at some point they’re still not having any symptoms and they say they’re fine then some will let them go back in. Some there’s protocols now, like not for the rest of the game depending on how significant it is. But I’m not sure how often that’s done. And again, that’s self-reporting and a lot of kids they want to play. So it’s ‘nope, nope. I’m fine.’” (Parent# 31020) “If it’s not a great ref then sometimes [teams] won’t play by the rules or are more aggressive and don’t get called for it.” (Athlete# 22040). “I think sometimes winning takes over safety of children—and then so, you know, they might actually have a concussion, but nobody will actually acknowledge it because they want to make sure that they win.” (Staff/Coach# 23006) “There’s so many rules in place for middle school—like oh you can’t do that, you can’t do that cause they might get hurt. Well why allow it in college? It seems like if it was fine in high school it should be fine in middle school. So I think there is a little too much rules.” (Athlete# 72013)	

#### Participation

The category of “Participation” was present in athlete and parent interviews. Athletes noted discovering sports and playing particular sports that their family and friends had and were playing. One athlete noted that because his dad “was always really big on things like baseball and football and all that… I just kind of got into it really quickly and then continued it” (Athlete #12022). Athletes also noted that it was fun and a way to build connections with other people. Parents emphasized their desire “to make sure that… physical activity was part of how [their children] knew the world to be” (Parent #11056), and their hopes that sports would help their children “learn a lot of team type skills” (Parent #21023) to help “navigate some of the tumultuous transitions of adolescence” (Parent #11056).

#### Role/engagement

Parents and staff offered perspectives about their roles and engagement. Parents noted the time commitment required for their children’s sport participation, with one noting “depending on the season, it’s their whole life” (Parent #31004). The amount of time dedicated to sport practices and games increased when considering club/travel teams as well. Parents’ in-person engagement included dropping off and picking up their children at practices and attending a few games. In some cases, parents would help supervise events or get food for away games.

Parent-and-coach interactions varied school by school, and sport by sport. Parents and staff both acknowledged that although some coaches have been with the MS for many years, other sports struggled to find coaches annually. Some noted that coaches were still being sought weeks and days before the season started. In some cases, coaches were not MS staff, but rather, outside community members that were brought in “from the community, either from [nearby university] or someone who worked in the area or whatever” (Parent #31020). Because of the potential turnover in coaches, parents and staff both noted that establishing a sports culture within a school and within a team was difficult. However, the continuity between coaches and their athletes was important in building relationships. To help maintain contact, parents noted coaches providing their cell phone numbers, emailing parents on a listserv, and using third-party apps. Parents also discussed “team parents” that could serve as a point-of-contact between the coach and the parents.

Last, MS staff noted the duality of being teachers and coaches. Overall, there was a need to be a “teacher first, coach second” (Staff/Coach #13003) and “worry[ing] so much about educating the children” (Staff/Coach #73003). Thus, they relied upon athletic directors and the policies and procedures to help them to ensure the safety of their athletes. As one staff member explained, “the culture needs to be established up top and fall down” (Staff/Coach #53005).

However, school nurses were conflicted about the parameters of their role as a nurse in the school system as related to sport and injuries sustained after regular school hours. Nurses discussed the limits in their roles, emphasizing one-on-one care to students and if needed, education for parents. Nurses noted staff education was beyond their role. In regard to injury, nurses felt they had to “[follow] what the MDs are saying to do” (Staff/Nurse #44001) and notify the coach and parents about the need to seek medical care.

#### Competitiveness

The competitive nature of sports within MS settings was discussed in multiple contexts by athletes and parents. First, athletes and parents stated that MS sports could be competitive in nature, with one parent noting that MS football “is as competitive as the [National Football League],” and that the athletes “were interested in winning than anything else” (Parent #21006). Another parent was concerned that “over time…sports have gotten more competitive for kids, a lot more competitive, even like club sports” (Parent #31020). Still, athletes noted that MS sports’ competitiveness paled in comparison to that of club/travel teams. Parents also generally agreed, with one summarizing that MS sports were about “bragging rights” in which “people want to win and at the end of the whole thing, one team wins the whole thing;” in club sports “you get placed at whatever level. And then they go out and play all different types of games, sometimes tournaments in other states and things like that. So, in some ways it’s more competitive” (Parent #11019).

Parents also shared that the competitive nature of MS sports also depended on school- and sport-specific aspects, such as how strong a team was within its division. For example, one parent shared that at their child’s school, the junior varsity girls’ basketball team was highly competitive as its team won many games, whereas the varsity team was not. Parents also noted that certain sports such as cross country allowed anyone to join; however, other sports such as basketball and soccer would include tryouts and cuts, or only play certain players throughout games, which may contribute to the competitive nature.

Despite the competitiveness that may have existed in some of the MS sports, athletes still enjoyed playing because “it’s a way to get your emotions out and a way to work out” (Athlete #12002). One athlete noted that despite not winning many games, “it was just [them] having fun” (Athlete #32015). Parents also highlighted that certain teams were able to allow students to build rapport with other teams, through emphasizing fun over winning and ensuring teams cheered for one another.

However, when competitiveness was high, athletes sometimes felt it took away from their enjoyment of the sports as “it wasn’t really fun to like be there socially because everybody was just focused on…practice, practice, practice” (Athlete #12051). Parents noted that this could potentially place athletes at risk of sustaining an injury such as a concussion. Athletes agreed, noting that teammates played “a little bit more aggressive” (Athlete #12045), but that concussion was “really only talked about if it’s a competitive scenario” (Athlete #12002); in addition, athletes reported that their teammates may not want to report injuries because “they wouldn’t want to sit out of practice or sit out of a game” (Athlete #12051).

As a result, parents felt it was important for schools and staff to help set the tone for the level of competitiveness that would be permitted within the sport culture. Parents noted coaches were important in “bringing a positive spirit” and when coaches were not focused on this, “it set the tone for the kids. It show[ed] what they [were] focusing on. It show[ed in] even what skills and drills they’re doing” (Parent #31020).

#### Safety

Athletes, parents, and staff all noted the role of safety in the MS sports culture. First, it was difficult to establish a safety-focused sports culture as coaching was a volunteer “side gig” that was not a full-time job, and the school districts did not employ athletic trainers. With concussion specifically, some parents believed their children were not at risk and it was difficult to discuss as it was an invisible injury; parents were “more concerned about, like the tangible injuries, like torn ACLs, broken ribs, broken arm” (Parent #41003). Further, parents noted that youth athletes would likely underreport injuries because they wanted to play. As athletes explained, getting injured during sports participation was inevitable, as “you’re going to get hurt eventually sometime in the season on way or another” (Athlete #12002).

With that said, coaches were optimistic that the culture was slowly changing for the better and that the “the prevention aspect has definitely increased a lot” (Staff/Coach #43035). Others acknowledged that despite sport safety varying from school to school, knowledge gain had spread across schools and there was an emphasis on “always promoting the welfare of the student-athlete” (Staff/Coach #53003). Parents, athletes, and staff all noted various parties within MS sports as influential in helping to contribute to safety norms. Athletes discussed being taught by their coaches on how to play their sport safely and properly. Parents noted that “safety [was] the priority over winning and really over anything else. As parents we should demand that” (Parent #11056)]. One coach emphasized that “the refs need[ed] to take the forefront” (Staff/Coach #73003)]. And a parent stressed that “a sport safety culture you know does come from a coach, but also from the school and kind of the community” (Parent #21023).

MS sport groups also described the need to be respectful of rules, such as understanding the need to stop plays when they hear the official’s whistle and avoiding foul play to avert injury. Parents and staff mentioned that the implementation of policy could help encourage safe play as well. Coaches noted their training, including CPR/First Aid certification. Policies were in place to ensure the reporting of concussions when they occurred. But parents described strategies to reduce concussion from “changing how they practice to reduce the occurrence of concussions” (Parent #11045) to removing heading in soccer.

Despite the confidence noted in coaches’ safety knowledge, all MS sport groups indicated concerns about how well policies were implemented, particularly when there was pressure to win. This included athletes feeling pressured to continue playing and thus not disclosing their injuries, officials missing illegal/foul play, and prioritizing winning over safety. Further, athletes noted that there were more rules in MS sports than what they saw at higher levels (e.g., high school, college, professional) and they were confused as to why such rules needed to exist (e.g., heading ball, tackling).

### Communication

Communication considered how MS sport groups talked with one another and what they talked about. Identified categories were pathways of communication, content, and mode, with school staff further clarifying on associated barriers to communication. Table 4 provides exemplar quotes related to the categories and sub-categories.

**Table 4:** Categories identified in interviews with middle school athletes, parents, and staff related to the category of “communication”.

Category	Example quotes
Pathways and content of communication between MS sport groups *Athletes (n = 19/19)* *Parents (n = 20/20)* *Staff (n = 18/18)*	*Coach discussions with athletes about fair play* “The coaches might say be cautious and respectful of others. And try not to be super aggressive” (Athlete #12022) “trying to teach them in addition to [their sport], how to be a good citizen, those kinds of things.” (Parent #11019) *Coach discussions (or lack there of) with parents* “Depends on the coach. I wouldn’t say they communicate with us a lot, it’s mainly around sort of the logistics—of getting to the right place at the right time.” (Parent #11056) “…there were a couple times I had face to face conversations with the coach. And it was at the beginning, just introducing myself and you know, letting her know that I was excited about <<Athlete’s Name>> being on the team.” (Parent #21023) “We don’t really have, I guess, more personal conversations with parents unless they initiate that.” (Staff/Coach #23001) “I have not talked with school staff. Really haven’t talked with parents a whole lot. Again, mostly just the athletes about things on the court and in practice.” (Staff/Coach #43003) *Discussions of injury*, *particularly concussion* “If there’s like an injury I try to send a notification to an individual parent-”(Staff/Coach #33003) “We just said said person had a concussion, was going to be out to be out for a while and then we moved on.” (Athlete# 12056) “You always hear like somebody got a concussion that’s why they can’t play or—But not that much.” (Athlete #22040) “I guess, we usually start by just—it usually comes up with an incident. So, we kind of describe what happened in the incident. Honestly, we don’t really talk a whole lot about prevention of it because when we’re talking about it, it’s kind of already happened. And so, the conversation is more, what we should do moving forward. And then maybe when the athlete starts playing again, we start talking about things to do on the court to avoid reinjury.” (Staff/Coach #43003) “It does happen. Well, like I said, we have our preseason meeting. And those topics are addressed and discussed… seldom do we get any questions about this. So it’s hard to kind of answer anything if they’re not inquiring. During the season, if it does happen, then obviously we’re handling it case by case. Postseason, we really don’t talk much about it so, just get ready for the next season. That’s kind of what we’re doing.” (Staff/Coach #53003) “We have an interest meeting for the kids at school and you’ll have a parent meeting usually after the first week of practice and after that you really don’t have much of, it’s really not conversation time. It’s more worried about—- Play and practice and learning how to play the game. So, that can be an issue. I think we try to impart in our players, because once an injury happens we talk about it.” (Staff/Coach #23003) “I remember talking about concussion and I didn’t really feel qualified to talk to a kid about it” (Parent #21006). “I mean I think it’s a…it’s a conversation you need to have or you can have it in the proper setting. You know, it’s not just one of those ‘Hey, I’m going to grab this out of left field and start talking about concussions. . .’ So, whether…there may not be a good opportunity that presents itself to talk about it. I mean obviously, if you’re in a sports situation you’re going to have to play football…or watching your kids playing football, you can bring it up. But if you’re out having dinner with a bunch of people or something, I don’t think, you know, concussions is going to come up a lot.” (Staff/Coach #53005) “I certainly wasn’t going out and saying, ‘Hey, my child had a concussion and here’s what he or she struggled with, has your kid ever had that?’ But it’s amazing how many people just start talking” (Parent #11051) “So it kind of wasn’t—it was talked about I think with one player because he actually had a concussion, but with the other players and like during the plays it wasn’t really talked about much it was just more of like, what can we do to provide … support” (Parent #41003) *Parent interaction with one another* “With the parents it’s usually like at the games, the ones who come to the games are sitting by each other and cheering together, talking together. At the end of the season we generally do some sort of end of season party in an altogether different setting than a game that is celebratory.” (Parent #11022) “We [[the parents]] all want to know each other and talk about the game and compliment their kids on how they’re playing and what not and other feedback or nice things you’ll mention about their son type of thing. So, yeah, we’re always happy to see each other at the games and you show up and it’s “Hey, there’s parent x and parent y.” We sit together and just kind of talk.” (Parent #21006)
Mode *Athletes (n = 8/19)* *Parents (n = 18/20)* *Staff (n = 16/18)*	*Use of school athletics website*, *apps*, *or email* “We have like a big communication system—called 8 to 18 that we all kind of like communicate through and emails, like we have a weekly email that kind of like settles out where all the fields are being used and all that and then like on the field pretty much.” (Staff/Coach #33003) “We have an App that we go through. Like a management software, it’s called Team Snap. And I mostly just send emails through that and it has the schedule and the volunteer slots on it. Oh, that’s how I communicate with parents on the team and players too.” (Staff/Coach #43001) “Most coaches here, do one of two things. If they are a bigger team—like a non-cut sport. Like track, or cross country, they have easily 60 athletes. They use Remind. Our smaller teams use email contact for the most part.” (Staff/Coach #73003) “But then what the coach does is…he sends out a weekly email. Each week he’ll send out ‘Hey, this coming week we have this game here,’ ‘don’t forget about this thing”‘ or whatever. And then he’s like ‘email me any time if you have questions or whatever.’ That’s the best way.” (Parent #11019) “The 8 to 18… I’m getting used to it. You know, it’s different because it’s, you have to like work to get to it…” (Parent #21040) *Role of a team parent* “And if you don’t have a parent who’s going to monitor and manage that it doesn’t always work well. If you have a parent who steps up to be sort of an administrative person who can stay on top of that, the coach can reach out to you, for example, that works better.” (Parent #31015) “Yeah, we have a team parent. And the team parent usually relays other messages to the other parents.” (Staff/Coach #13003). *Communicating through the athletes* “But a lot of times our information is going through the athletes every day at practice. So we are hoping that they transfer that information to their parents.” (Staff/Coach #73003) *Preseason parents meeting* “We do have a parent meeting in the beginning so that sort of everybody gets the same general athletic information. And then sport specific information.” (Staff/Coach #73003) “We have our preseason meeting. . .We answer questions, Q&A.” (Staff/Coach #53003)
Barriers *Staff (n = 15/18)* *Not a category for athletes and parents*	*Athletes and parents not being receptive to concussion prevention and response messaging* “Like when people aren’t totally receptive—Like, you know the kind of, oh it’s just like, you just suck it up or something—if someone’s more like that it’s really hard to talk.” (Staff/Coach #33003) “I think sometimes people are just like…we have this mentality of just like ‘man up’ or ‘just push through it’ kind of. And so….I don’t think people fully understand the full effects. Again, like the long-term effects of having concussions. So, I think sometimes they just brushed it off. Like, ‘that’s not really like a big deal.’” (Staff/Coach #73010) “Yeah, and it’s also that they’re in middle school and they’re stubborn and sometimes they just don’t realize it and they think they’re invincible and it drives you nuts. I mean I’ve heard stories, [inaudible] my stories of kids who just are ‘Oh, I’m fine.’ ‘You’re really not.’ And they just think they can go through it and it’s ‘You need to tell me immediately when it happens because that’s the best thing for you. Don’t make it worse than it is.’” (Staff/Coach #23003) “I think it’s kind of that balance between like, not wanting to freak out the parent, but also wanting the parent to take it seriously. You also, you know, run into parents who will say, you know, ‘my child is fine’ and you really disagree with them. So that just gets kind of gets uncomfortable.” (Staff/Coach #43003). *Parents not trusting expertise of school staff* “You’re looking at the <BLINDED> school system that has a very high rate of highly educated parents that may not want to hear anything less than a physician. You have another population that a nurse might do it. You have a population that’s like “Yeah, I don’t want either one of you.” (Parent #34001) “Maybe with a parent after a kid was injured. And like they’d be accusatory looking at me, like, why didn’t you stop this from happening? …you know, questioning my coaching. Rather than, you know, why the kid got hurt. Obviously, I can tell you why their kid got hurt, but if a parent was upset with the way it happened and tried to maybe spite me for something like that, would be uncomfortable.” (Staff/Coach #43004) *Length of season* “with the middle school sports season being so short, if they are experiencing a concussion in the middle, they are possibly out for the rest of the season” (Staff/Coach #44001). *Communication issues* “If a concussion happens at home, on vacation, etc. I may not find about it for a while. Because if the parent doesn’t let us know we won’t know. Sometimes later the child will be having symptoms and the parent might let the coach know and then they can let me know.” (Staff/Coach #14001) *Barriers related to athlete’s homelife* “We do have some English language barriers, parents for whom English is not their first language. It’s typically Spanish. We have a Burmese girl this year on our team too. Her mother is from Burma so there is a language barrier. (Staff/Coach #23033) “I can’t tell you how many different countries, how many different languages. So, sometimes culture will play a role in what they’re willing to do specifically with the medical community.” (Staff/Coach #44001) “Some kids don’t even have a parent, right? They’re foster care.” (Staff/Coach #34001)

#### Pathways and content of communication among MS sport groups

Numerous pathways of communication among MS sport groups were noted by athletes, parents, and staff, with content varying with each pathway. Most athlete conversations were with one another about sports, be it their own games or the professional and college sports they watched. Athletes stated that their coaches led the conversations on gameplay, skill development, in-game rules, and general safety. Concussion was rarely discussed among teammates and mostly by coaches and parents. Numerous athletes noted that most discussion occurred “towards the beginning [of the season], then you don’t really talk about it unless it happens” (Athlete #22040). Parents also acknowledged that coaches emphasized “trying to teach… how to be a good citizen” (Parent #11019). Coaches added that conversations focused on logistics, including scheduling and transportation needs, or if they needed to disclose a child’s injury. Both parents and coaches admitted that otherwise, it was parents who had to initiate conversations. Coaches also rarely spoke with one another unless it was during meetings led by the athletic director or with their assistant coaches.

Parents also noted the struggle in interacting with athletes aside from their own children as they had to work or were spectators at games. In addition, parents disclosed that when an athlete on their child’s team sustained a concussion, it became an important discussion point among one another. Parents would check in with one another and try to find out how to be supportive to the concussed athlete and their family. However, they noted feeling awkward in discussing concussions because they did not “really feel qualified to talk to a kid about it” (Parent #21006). Last, parents noted that communication among one another was fragmented and based upon friendship circles built outside of the sport. With that said, parents were open to socializing at games and other events provided by the schools, such as complimenting their children’s’ improvements in game play or attending end-of-season get-togethers.

#### Mode

MS sport athletes, parents, and staff had varying views regarding the modes of communication used and their utility. Athletes talked about using various modes of communication in their interaction with other athletes, including social media, email, and text messaging. However, they noted not really paying attention to any official communication from the school, and that most conversations with teammates were more about how games or practices went and any injuries that may have occurred.

Parents and coaches noted various modes of communication that allowed them to keep in contact with coaches. Schools had different official means of communication, such as through the school athletics website or apps such as “8 to 18” or “Team Snap,” although this varied school to school and sport to sport. Parents acknowledged struggling with access to these resources or limited information being provided. They noted that some coaches seldom communicated through the website or app; coaches themselves disclosed email being a more preferred mode of communication. Parents also admitted they were prone to forgetting to check emails regularly. As a result, parents identified start-of-season parent meetings and speaking with coaches before or after games and practices as viable communication options. Parents also relied on obtaining information from their children directly or a designated “team parent” who could “relay messages to the other parents” (Staff/Coach #13003).

#### Barriers

Last, school staff were asked about any barriers to communication they have with other MS sport groups. The most prominent discussion point related to the struggle to communicate with athletes and their parents. Coaches discussed athletes thinking they were not at risk for concussion and struggling to retain any educational information provided to them.

When communicating information with parents, coaches had to consider many different barriers. These included parents who were indifferent to injury and expected their children to play through injury, or who did not notify the schools when a concussion occurred outside of the MS sport setting. Some coaches disclosed that since they were at schools with “a very high rate of highly educated parents, that [parents] may not want to hear anything less than a physician” (Staff/Coach #34001). However, coaches also had to consider manners of communicating so that they would not “freak out the parent, but also [have] the parent to take it seriously” (Staff/Coach #43003). Coaches were also concerned about parents blaming them when concussions occurred, or when athletes took longer to recover. Coaches noted that “with the MS sports season being so short, if they are experiencing a concussion in the middle, they are possibly out for the rest of the season” (Staff/Coach #44001). Staff noted that MS families may face barriers due to their living situations, including children being in foster care, language barriers, and cultural differences.

Finally, several staff noted the roles of traditional and social media in shaping parent perceptions. Television shows and movies regarding sports and concussions (e.g., “Friday Night Lights,” “Concussion”) focused on the risks of participation in contact sports, causing parents to worry about their children participating in contact sports. However, one coach noted that such media and the resulting discussion on social media has “helped take the taboo off of it and made it, people more comfortable to talk about it” (Staff/Coach #23006).

## Discussion

In this qualitative study examining sport culture and communication among MS athletes, parents, and staff, overall findings suggest there may be factors within MS sport culture and communication that delineate it from other amateur sport settings, such as high school and collegiate sports, and youth/recreation leagues. In general, there was an emphasis on both athletes exploring the varied sport options offered at this MS, alongside engaging in fair play and sport enjoyment. However, participants expressed sport safety, particularly concussion prevention, was historically not of a high concern, but that attitudes were changing. In addition, communication among MS sport groups focused primarily on logistics (e.g., schedule changes) and relied on various modes of communication, which created challenges for parents. Overall, the findings highlight the need for education and prevention interventions that are sensitive to community needs. These interventions should consider not only the barriers to effective communication regarding injury but also factors emphasizing the potential severity of sport-related injury in MS athletes.

The participants interviewed from the MS sport settings noted an emphasis on athlete fun and the exploration of sports. Some parents noted this setting being less competitive and intense than youth/recreational league settings, and an opportunity to sample sports. These characteristics align with the Aspen Institute’s Project Play Initiative, which aims to identify manners to ensure all youth are exposed to and can participate in sports and physical activity [7]. In particular, Project Play emphasizes asking children what they want to play and discouraging early sports specialization. The latter recommendation is especially important given research suggesting early sport specialization is associated with increased injury risk [33], although calls for continued research to fill knowledge gaps exist [34]. Given declines in youth sport participation in the past decade, it is essential to identify manners to ensure opportunities for sport participation, particularly as it has been associated with positive health effects [1]. With that said, there was variability in how participants perceived sport culture, as some noted great competitiveness. Thus, such efforts to encourage youth sport participation need to consider the different perceptions associated with youth sports.

At the same time, MS sport settings contain challenges often not present in other youth sport settings. For example, schools represented in our sample often struggled with frequent coach turnover, with some instances of identifying, recruiting, and hiring coaches near or at the start of the sport season. Further, sport seasons were short in duration with injuries potentially causing an athlete to remain out for the remainder of the season. As a result, it may be necessary to include and integrate outside support and resources to help establish and nurture a healthy sport culture, perhaps at the overall school level or at the district level. From a socio-ecological perspective [35, 36], athlete health and safety is affected by numerous levels of influence, all of which may need to be considered in injury prevention. Previous research has noted the importance of parents and coaches in the development of youth athletes and their safety [13–15, 18–22]. However, additional groups of individuals within MS sports and within the schools overall should also be considered, including sporting officials [37], teachers, principals, and district superintendents.

Also, as noted by a report from the Aspen Institute [38], a number of youth sport coaches are not trained in general safety and injury prevention, concussion management, and skill development. Consequently, certification and resources from national organizations may help to nurture and enrich this culture of safety as well. Further, sport-specific organizations (e.g., US Soccer Federation, US Lacrosse, USA Hockey, USA Football) have developed athlete development models to help organizations and coaches ensure appropriate game play and skill development [39–41]. MS may benefit from utilizing such resources to identify and implement age-appropriate sports curriculum. Last, given that sports injury prevention mandates and recommendations may not always be applied as intended [42–44], it is imperative to focus on identifying strategies to aid proper implementation.

Communication difficulties were also noted by the MS sport groups. In some cases, each school (and then each different sport within each school) had various mechanisms of communication and some struggled to keep up with emails and messages from the utilized apps. Further, communication from coaches and school officials mostly pertained to logistics and had little discussion pertaining to injury prevention. It is possible that MS in general may struggle with communication with parents. Nonetheless, the findings highlight the need to consider opportunities to elicit first, reliable and consistent means of correspondence among MS sport groups, and second, discussions about key athlete safety topics such as concussion recognition and management. Regarding correspondence among the MS sport groups, identifying appropriate communication may involve the need to better identify appropriate modes of communication that are more accessible to all parents and their athletes within MS sports. This may include consolidating into specific mobile phone apps, the use of a team parent or opinion leaders who are well-liked and well-respected, or a combination of these resources [16]. Regarding athlete safety, MS settings may benefit from communication that uses specific key messages and then integrates them into ever present aspects of sports play [37]. This can include the use of pre-game huddles that emphasize athlete safety [37, 45]. Most importantly, such communication must acknowledge any additional barriers that place certain athletes and families at risk of not having access to a certain mode of communication and/or not receiving appropriate messaging.

Moreover, additional barriers must be considered in order to ensure prevention and management strategies are feasible. These include language barriers that hinder parents’ ability to understand injury prevention and management information, as well as ensuring role clarity for school nurses in the management of concussed athletes, particularly with the absence of on-site athletic trainers, which are generally available during sanctioned sports at higher levels of play (e.g., high school, college). With that said, although our sample included seven MS from two school districts, it is likely that additional barriers are present in MS that may vary from our study sites, such as geographic location and socioeconomic status. This is especially important in the context of our study given that we only consented those who could understand English.

Finally, as this study is part of a larger project focused primarily on concussion education and prevention [16], the categories discussed should be considered in a similar context. Further, despite the comparability in concussion incidence between MS settings [8, 9] and other sport settings (such as HS, youth, etc.) [46, 47], research indicates significant concussion-related knowledge gaps among individuals within MS sports [48, 49], which may be perpetuated by a lack of prioritization as indicated within the findings presented herein. Concussion-related discussion elicited from the participants during the interviews highlighted a culture in which concussions may not be prioritized until they occurred. This likely originated from athletes not wanting to lose playing time due to injury [35], and a lack of understanding of concussion–particularly as they were harder to identify than other acute injuries such as sprains and fractures. Public health has generally discussed prevention in three stages: 1) primary, or preventing the adverse outcome before it occurs; 2) secondary, or aiming to identify and intervene on the adverse outcome as quickly as possible; and 3) tertiary, or treating and managing the adverse outcome after it has occurred in order to mitigate further associated complications. Although our participants noted recent efforts for primary prevention (e.g., coach training, reducing contact in practices), most prevention has appeared to be tertiary in nature. In addition, participants noted uncertainty in when and how to discuss concussions with others. Communication about concussion typically occurred solely in preseason meetings and when they were known to have occurred. Also, school staff were not sure about their roles when related to concussion, with nurses in particularly noting that they were not comfortable to go beyond their assigned roles and override recommendations that may originate from a doctor. These findings regarding communication parallel recent research that found that adults within youth sport settings believed they played a role in concussion prevention, but were uncertain and felt unsupported in how to properly engage with youth athletes to discuss it [50].

These findings highlight the need for injury- and concussion-related prevention interventions in MS sport settings that can prioritize primary prevention while nonetheless advocating secondary and tertiary prevention. However, these interventions should consider the unique aspects of the MS setting and structure interventions around these attributes such as the diversity of individuals involved in MS sports and limited resource availability. Importantly, they should help such individuals understand their role in promoting these prevention strategies and how to feasibly, yet effectively, participate as well. Moreover, to help reinforce intervention core components, the intervention should occur throughout the entire MS school year (and not just solely within a sport season), include multiple touchpoints [e.g., different times of the academic year (fall terms, spring terms, etc.)], and consider multiple modes of delivery (conversations, seminars, handouts, etc.).

### Limitations

As with all research, this study has its limitations. Our target sample only included seven MS in two districts in one county from a southeastern state. These schools may not sponsor sports available at other MS in the US. Further, within these schools, we relied on a convenience sample of athletes, parents, and staff to share their experiences. As a result, findings may not be generalizable to all MS sport settings. Also, as this study was part of a larger project focused on developing an intervention to help athletes, parents, and coaches in MS sports better communicate concussion-related prevention and management information to their peer networks [16], the semi-structured interview protocol focused primarily on understanding school- and sport-related factors related to education, safety, and communication. The included questions may have inadvertently limited the content and resultant categories identified. Nonetheless, this study provides insight into the unique circumstances associated with sport safety and injury prevention in MS sports and has the potential to aid the development of future research focused on this setting and interventions to mitigate the incidence and severity of sport-related injury among MS athletes.

## Conclusion

Interviews with athletes, parents, and staff highlight that the MS sport setting is a unique setting that may require novel and tailored approaches to injury and concussion prevention intervention to help ensure buy-in and proper implementation. Identified categories and themes highlight the sport culture and communication that exist within MS sport settings. Our findings suggest that MS sports may struggle with incorporating primary prevention into their cultures and ensuring reliable communication among individuals within MS sport settings. This study provides potential areas for further examination within the contexts of culture and communication that can hopefully help to improve this setting’s ability to help reduce the incidence of sport-related injury and concussion. This includes the development of prevention strategies pertinent to the setting, as well as understanding the manners in which they are implemented properly.

## Supporting information

S1 Checklist (DOCX)
