# Supplementary figures and images for: Sport culture and communication among middle school athletes, parents, and staff: A qualitative study
Source: PLoS One. 2023 Mar 15;18(3):e0282252. doi: 10.1371/journal.pone.0282252 (PMC10016647; doi:10.1371/journal.pone.0282252)

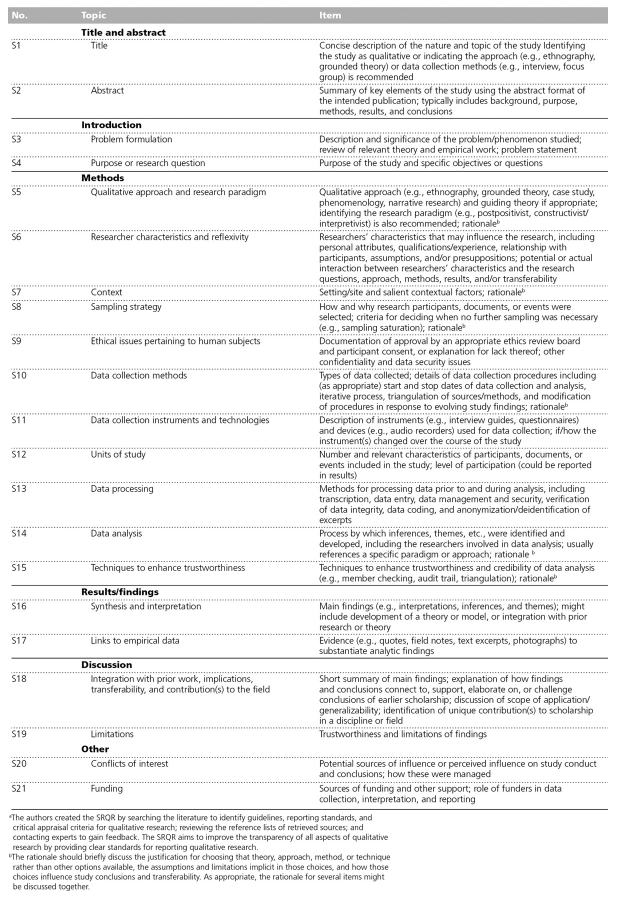


X

X

X

X

X

X

X

X

X

X

X

X

X

X

X

X

X

X

X

X

X

Supplement: S1 Checklist — (DOCX) [file pone.0282252.s001.docx]
